# Family and carer experiences of advanced care planning processes and outcomes

**DOI:** 10.1017/S1478951524001603

**Published:** 2025-01-23

**Authors:** Gail Whiteford, Dan Curley, Graeme Mooney, Leigh Kinsman, Tony Lower, Megan Hobbs

**Affiliations:** 1 School of Allied Health and Exercise Sciences Charles Sturt University, Lake Innes Forecourt, Port Macquarie, NSW, Australia; 2 Mid North Coast Local Health District, Coffs Harbour, NSW, Australia; 3 La Trobe University, Bendigo Campus, Bendigo, Australia; 4 University of Sydney, Camperdown, NSW, Australia; 5 University of New South Wales, NSW Australia

**Keywords:** Advanced care planning, end of life care, advanced care directive, carer, qualitative research

## Abstract

**Objectives:**

Despite practice development in the area of advanced care planning (ACP) and systems wide changes implemented to support ACP processes, there has been a paucity of research which has addressed the experiences of a key stakeholder group – family and carers – as they navigate their way through these often very challenging processes. The study described in this article focussed on this key group.

**Methods:**

In-depth qualitative interviews were undertaken with family members and carers in a regional area of Australia in order to illuminate their lived experiences of ACP processes.

**Results:**

Thematic analysis of the narrative data yielded 4 key themes: Being overwhelmed on the ACP journey; unifying effects of completing and using an advanced care directive (ACD); experiencing the highlights and lowlights of care; and paying it forward in advice to staff, carers and families.

**Significance of Results:**

The ACP journey is unique for each carer/family and can be overwhelming. Whilst he lived experiences of families/carers indicated that the quality of care received was of a high standard, feedback to staff suggested their communications be timelier and more empathic. All participants in this study reported benefitting from engaging in ACP early and appreciated support to do so. All benefitted from the preparation of an ACD and found the outcomes (in terms of concordance) gratifying.

## Introduction

A broad definition of advanced care planning (ACP) is that it is a co-ordinated process involving terminal patients in future decision making about their care through ongoing discussions and review cycles (Blackwood et al. 2019; Dixon et al. 2018; Schichtel et al. 2019). ACP has tangible artifacts with a procedural orientation including, ideally, an advanced care directive (ACD). Indeed, the presence of ACDs and ACP documentation is often used as an outcome measure to provide an indication of alignment between stated wishes and actual events (Schichtel et al. 2019). Importantly though, as well as understanding ACP processes from a purely procedural perspective, it is crucial that ACP processes are understood as a vehicle for dialogic interaction between all stakeholders (Rietze and Stajduhar 2015; Risk et al. 2019) and as constituting a psychological/emotional “rite of passage” for the patient (Hole and Selman 2020; Lund et al. 2015). As ACP involves patients discussing their healthcare preferences and wishes with family, carers, and providers, it is also an important guide for future treatment if, ultimately, the patient has diminished capacity (NSW Health 2013). It should be noted that ACP differs from *Shared Decision Making* because it focuses on future decisions and anticipated deterioration (Blackwood et al. 2019).

Generally speaking, ACP is regarded by health professionals, carers, and patients as having a positive impact on end of life care (EoLC) internationally (Higel et al. 2019; Waller et al. 2017; Weathers et al. 2016). More specifically, ACP is viewed as being able to: increase compliance with patients’ treatment preferences (Detering et al. 2010; Martin et al. 2016); reduce admissions to aged care facilities (Brinkman-Stoppelenburg et al. 2014; Gleeson et al. 2019; Martin et al. 2016); reduce unnecessary hospitalizations and deaths in hospital (Gleeson et al. 2019; Lund et al. 2015; Martin et al. 2016; Molloy et al. 2000; Weathers et al. 2016); and, reduce the psychological distress experienced by bereaved family members (Brinkman-Stoppelenburg 2014; Detering et al. 2019; Lund et al. 2015; Trankle et al, 2020). ACPs have also been linked to shorter length of stay for people presenting from residential aged care (Street et al. 2015).

Despite such benefits, difficulties remain for health professionals (including GPs) engaging in ACP with patients (Risk et al. 2019). Several reasons for this have been proposed including, primarily, that they may feel they lack knowledge, education and confidence in how to facilitate ACP conversations (Batchelor et al. 2019; Blackwood et al. 2019; Fletcher et al. 2016; Risk et al. 2019). Additionally, ambiguity may exist among healthcare workers about whose role it is to initiate ACP discussions and whether those discussions are within their professional scope of practice (Rietze and Stajduhar 2015; Risk et al. 2019). In some settings, clinicians may be required to self-determine their roles around ACP, which may only compound ambiguity in practice (Fletcher et al. 2016). Additionally, a significant factor preventing consistent practice in this area is that health professionals lack adequate time to undertake ACP in some clinical environments (Batchelor et al. 2019; Blackwood et al. 2019; Rietze and Stajduhar 2015).

For patients involved in ACP, it can be a challenging process that needs to be unequivocally person-centered (Hopkins et al, 2020; Rhee et al. 2012). Indeed, what is requisite is clear, jargon free, information to enable them to make values-based decisions, especially with respect to EoLC. This is especially important as people who become terminally ill patients come from diverse backgrounds and have varying levels of health literacy and literacy in general (Risk et al. 2019). Reflecting such diversity, the way in which patients respond to ACP discussions can be varied (Lund et al. 2015). People may be hesitant to talk about the process of dying (Mignani et al. 2017; Sellars et al. 2018) and, as a corollary, staff may be reluctant to facilitate the discussion for fear of negative impacts (Blackwood et al. 2019; De Vleminck et al. 2013). For some patients, there can even be a denial of the relevance of ACP processes despite advice to the contrary (Rhee et al. 2012; Schickendanz et al. 2009).

As for the final key groups involved in the ACP process – family members and carers – the available research on their experiences and perceptions can be described as relatively “patchy.” Some research points to ACP “hesitancy” (Batchelor et al. 2019; Mignani et al. 2017) whilst on a continuum of experience, others suggest that ACP is experienced as a positive collaboration (Carrasco et al. 2021). In contrast, an RCT undertaken by Molloy (2000) indicated that family members found the process of little or no value. Clearly, there needs to be more research in this area which systematically focusses on family and carer experiences to inform best practice and future policy development. Whilst there have been some qualitative studies in this arena (see, for example, Hossain et al. 2022; Kupeli et al. 2019; Meeker et al. 2014, and also; Weindrich von Dael 2020’s umbrella review) the need for more qualitative exploration is indicated in order to gain greater insights into the ACP journey as a whole and to illuminate the lived experience of families and carers throughout that journey.

## Methods

### Setting

The study described in this article took place as part of a large, multi-method intervention focussing on ACP processes and experiences as well as on concordance between ACDs and outcomes across multiple community and hospital sites in the Mid North Coast Region of NSW. Following Ethics Approval from NSW Health (no 2019/ETH11902) the large study took place over a period of 2 years (within the COVID restrictions period) between 2020 and 2022 with the qualitative sub study described here undertaken during 2021.

### Participants and recruitment

Participants in this study were family and carers of patients who were in palliative and EoLC. The majority were home based and supported by community health workers and GPs, but with some episodes of care being delivered in hospital settings on an urgent/needs basis. The patients, family, and carers who were part of the large study which supported them through ACP processes (including generating and uploading an ACD) were all provided preliminary information by nursing and social work staff about the qualitative study when they conducted home visits to them. After a period of 6–12 months following the death of the patient, family and carers were once again provided with information regarding the qualitative study and given contact details if they wished to participate. Six family members/carers of the total intervention cohort made contact in order to arrange an interview with a research team member with whom they had not had any contact previously.

Following informed consent processes, participants were interviewed during May 2021 using a semi structured interview schema (approved by the Ethics Committee). Each interview lasted up to an hour and was conducted by phone due to COVID related restrictions at the time. Transcripts were generated through use of NVivo transcription software. These were then assigned code names and any identifying features were removed.

## Data analysis

The analysis of the narrative data generated through the interviews was theoretically informed by the *qualitative description* approach. This approach, relatively new in the qualitative/interpretive tradition, has been described as a relevant approach where:

… information is required directly from those experiencing the phenomenon under investigation, where time and resources are limited and perhaps as part of a mixed methods approach (Bradshaw et al. 2017, 1)

It should be noted that one of the additional benefits of a qualitative description approach is that it allows the researcher to be responsive to data as it emerges rather than being committed a priori to a particular theoretical framework (Sandelowski 2010). Analysis then involved an open coding process (Saldana 2016). The coding process used a manual, in-vivo coding approach which foregrounds the everyday language of the participants (Creswell and Poth 2014). Codes generated were then clustered together into emergent themes (Liamputtong and Ezzy 2005) using visual display methods (Maher et al. 2018). These emergent themes were then discussed, refined and consolidated among the research team. Final themes generated from the analysis process were:

1. *It’s overwhelming*: Reflections on the advanced care journey
2. *It Unified the Family*: Completing and using the ACD
3. *All happened seamlessly/An overloaded system*: Highlights and lowlights of care
4. *Anything we can to pay it forward*: Advice to staff, carers, and families

These themes and supporting narrative data extracts are presented in the following section.

## Results/Findings

In the first theme, participants shared their reflections on the whole process that they had lived through with the person that they had been caring for. Some spoke to the entirety of the process, indicating how demanding it was for them

*The whole process, its overwhelming, the whole thing’s overwhelming* (Ruth)

and times when it was difficult to cope and how, during the process, there was a need to be in advocacy mode

*I had blinkers on for a long time. There were things I didn’t want to see or hear, which I guess is fairly normal* (Sandra)

*I think you have to be a staunch advocate for yourself and your family* (Carol)

For others, there were specific events and developments that they particularly highlighted in their reflections. Some were more positive than others such as this commentary on the importance of supportive equipment provision

*I lived close to Mum but when she started getting in heaps of pain and needed things like toilet seats and walkers and a couple of ladies bought pieces of equipment to the house and another time we went to the warehouse and picked up some of the stuff, but whatever she needed, she got* (Anne)

Whereas for this participant, their anxieties around the provision of pain medication in the home setting are evident and appear to be a key event in their experience

*I’ll tell you one thing, I was scared of the morphine bottle they gave me in his medications … you know, you say the word “morphine” and you automatically think – a druggo, not being in the nursing profession … so I eventually rang palliative care back and she said “you can give him some of that every hour if you need to, it’s such a low dose” … but I was so anxious I was going to OD him, it’s my father, God, you know, and I’ve got his life in my hands* (Nerrida)

What is evident in the narratives is that there is not one homogeneous experience of EoLC and that carers and families can respond very differently depending on their specific sociodemographic and sociocultural contexts. In this narrative extract, the participant reflects on the moment in the journey when there was a realization that home based EoLC was not for them

*I looked after him for quite a long while but his health just gradually went downhill and he got to the stage where I had great difficulty doing things for him and palliative care came to see both of us and they said “are you managing alright? Can you carry on looking after Ed?” … but then they started talking about bringing in a hospital bed and having it in the lounge and I thought “oh no, I couldn’t handle that and I don’t think Ed can handle it either”* (Doreen)

In the second theme, which is about how unifying completing the ACD was, experiences were uniformly positive. Some started a little uncertainly in terms of not feeling ready to commence, such as in this story

*Someone gave us the Advanced Care Planning booklet, and I had started it, but Sam was a bit, well, like he didn’t want to, so, Id started it but thought “he’s not ready to do this” so I left it, then the Social Worker got involved and said “it’s a good time to get this done” and then he was talking to Sam too and we got it done (Ruth)*

Others however, focussed on the value of completing the ACD while the patient was well in order to ensure that care was consistent with the wishes of the patient.

*They did everything they said they would and we got the care that he needed as they said they would. Putting the advanced care plan in place was a great thing because I set it up and he had a say in it. We did it while he was well (Sandra)*

*I think it (the ACD) is important because the loved one needs to set down what they want because if they die unexpectedly and there’s no lead up to being ill then people say “oh my God, what am I going to do?” To me it’s important that they have something like that and that all the different people at the hospital all have a copy of the directive and they know where to look for it. It’s what Ed wanted and I can’t stress more strongly that they should fill out the form (Doreen)*

*I said [to the person from palliative care] I’m going to put the ACD on the TV stand, which is where you open the front door for when the ambos come in … it’ll be sitting there, its not moving, I think they found that good because it was right there and they knew exactly what they had to do [if an ambulance arrived] (Nerrida)*

In these final reflections, participants variously reflect on the unifying value to family of being involved in ACP, some of the challenges of actually completing and uploading the ACD, and on how the ACD has had an enduring personal impact.

*You need to get the whole family together and talk about doing the advanced care plan so they can see the importance of it (Nerrida)*

*We followed it to a T because we had it in black and white – that’s what Dad said and sometimes we’d call each other up and go, hang on a sec, we are not going to do it like that, we are going to do it properly, that directive was Dad’s voice up to when he died and after … it drove us as a family and it unified us (Carol)*

*I uploaded the ACD, it was easy, I mean, if you are computer literate it is easy but my mother-in-law or even sisters would probably struggle and would rely on their children – otherwise I don’t know how they’d do it (Sandra)*

*The staff explained it and helped us, there were only two questions we were not sure about. Doing the ACD was good though I think it was confronting for Mum, but for me, knowing what she wanted and what she didn’t want … I carried it with me in my wallet everywhere I went because if someone were not to do what she wanted then I’d have to [tell them] so it’s in my wallet right now, it’s been nearly six months and I haven’t taken it out yet (Anne)*

In the third theme, *Highlights and Lowlights of Care*, experiences of the healthcare system are the basis of the narrative accounts. In these accounts, the focus is on the quality of staff input and how the health professional staff families and carers interacted with, went out of their way to accommodate needs. It should be noted that some of the reflective accounts reference the COVID context

*Everyone was very good, I can’t knock any of them as Ed’s wellbeing was at the core, they really looked after him, they were just excellent, excellent … they didn’t poo-poo everything and say “you should be doing this, blah-blah” but they had everything in mind and came up with suggestions … [like] he had one of those chairs which you can manipulate and the back goes up and legs go out. He actually lived in that* (Doreen)

*Mum started to get scared [of being on her own] and the nurse said she could organise for me to get another bed in the room [at the Pal care unit] – she said, “I’ll make it happen right now” and got a second room ready with two beds and that night we started sleeping over every night. [One night] she called [about mum deteriorating] and said, “this is it” and [although it was COVID precautions] she allowed us all in and said “I’ll probably get in trouble for this but I will deal with whatever in the morning”* (Anne)

*The staff were brilliant. They were compassionate … and even with things like the oxygen and stuff, they organised all that which was great, and of course his medication, they were very helpful, when you’ve never been involved in anything like this, you don’t know what questions to ask and they were able to anticipate what I would probably need. They also made a couple of trips actually as Dad had trouble swallowing so they organised the dietician to come with water thickening stuff and showed me how to use it. They recommended different things that Dad could try – I had tried a few things but what were we down to on the second last day? Porridge and a beer! And she [the dietician] knew I was worried because I thought he was malnourished, but [I got] the little “pep talk” and you think “thank you!”* (Nerrida)

For this participant, however, whilst they point out that there are “wonderful people” in the system, its essentially “overloaded”

*The healthcare system for me - and I’ve journeyed through it with mum with Alzheimer’s -it’s a very overloaded system … it’s just a lot of people don’t take personal responsibility for what they can do for their own health. But anyway, for lots of reasons, I think it’s overloaded. My experience has been that you have some wonderful, wonderful people [but] it can be very easy to slip through the net* (Carol)

In the fourth and final theme or paying it forward, advice to other families/carers and staff, summative reflections are captured. Here, there are recommendations to other family members that they need to ask for help and be more assertive and also that they keep a journal

*Ask for help, ask for help and ask to see the social worker, ask for help if you are struggling. And another suggestion is try not to be alone because that’s awful … you know, family and friends are important, couldn’t have gone through that without my family and friends … and you need to be more assertive than I was at times* (Ruth)

*I did have a notebook where I was jotting down all the medical stuff that was happening but I look back and wish I’d written more about the emotion and feelings … because now when I look back, I feel like I’ve missed something … it was just such a whirlwind of days and I wish I’d written it down (Ruth)*

However, overwhelmingly, the sense of “paying it forward” in the form of advice (a term used by a carer) was directed towards the health professionals the participants had interacted with during the ACP Journey. At one level, the advice centered on the need for very practically oriented support, such as where to park and get food during a hospital admission of the patient.

*I would say bear in mind that the carer, like me, [probably] rarely goes to hospital, doesn’t know the routines, what floor things are on, you know, make sure they know where they are going and what they are doing – you know, don’t take the first car park because where you want to go is another kilometre up the road … you know, nobody told me there was a canteen, so someone needs to [give] a mud map* [Nerrida]

This extended through to more specific feedback (based on experiences with staff) focussing on clear, consistent and genuine communication and the need for health professionals involved to “not lose track of the soul” even when they are involved in EoLC care on a daily basis.

*It is easy to be intimidated by doctors and nurses. Sam couldn’t tell me what they had said because he may have been feeling too intimidated … I was there but it’s hard to catch them … I think they have to realise that they need to communicate with the family more (Ruth)*

*I would say, listen! Ask how they are, but sincerely, it’s very easy to detect non sincerity. It has to be genuine; it has to be someone who sees that as their line of work in life, who is somewhat passionate rather than just doing a job … the people who have made a difference are the people who don’t lose track of the soul – because the people they are dealing with are not machines* (Carol)

To close this section, we present here a moving narrative extract that speaks to the totality of the experience and the demanding nature of the journey through EoLC for family/carers. In this narrative there is an acknowledgement of the importance of the ACP process which, whilst positive, was nonetheless, very demanding. It’s a poignant reminder that even though the person they are caring for ultimately dies, it is not the end of *their* narrative.

*All I can say is that the care we received was fantastic in my book, having never been through it before and hope to never go through it again because it’s horrible. To watch the person you love disappear. I mean I was lucky, he passed away at home and that was what he wanted and everything was put in place to enable him to do that … I was lucky I also had family support and it makes a big difference, it’s better than passing away in hospital so, you know, having him have that input into the advanced care [plan] - what his wishes were so he could tell people himself what he wanted and how he wanted it, it was a great thing … but it’s hard for things afterwards, I battle it everyday*

## Discussion

There is no such thing as standard journey through ACP and EoLC for patients and their families/carers (Bischoff, 2013; Kupeli et al. 2019). Indeed, each journey is uniquely shaped by multiple, intersecting demographic and sociocultural factors. Based on the narratives of the participants in the study presented here, it seems that the journey is characterized by, at different stages: uncertainty; varying levels of support; feelings of being unready, overwhelmed and/or stressed; eventual acceptance and, for some, relief. It should be noted that, as the final narrative extract presented suggests, that even though there is an eventual death, the carer/family member(s) remaining can still struggle. As for the specific act of completing an ACD, all participants perceived it as being very beneficial. They reported needing and appreciating practical and emotional support from staff to complete it, especially online and viewed a need for more awareness raising of ACP processes in general, as has been suggested in other studies (Meeker et al. 2014; Weindrich-van Dael et al 2020). All felt that there had been concordance between the ACD and eventual outcomes (Tark et al. 2020).

Whilst the quality of care was overall perceived as positive, participants focussed more on individual staff per se with nursing staff, allied health and GPs being specially mentioned. Negative feedback in relationship to staff was most often pitched at specialists who seemed to have too little time for interaction and honest communication, and at system wide failings – reflecting a view that the health care system is “overloaded.” This is an interesting finding when, overall, time taken to facilitate and support ACP can actually reduce EoLC costs significantly (Dixon et al. 2015; Molloy 2000; Schickedanz et al. 2009; Zhang et al. 2009).

Summative reflections of participants suggested advice in the form of being assertive and seeking support. Specific feedback to health professionals stressed the need for empathic, genuine and timely communications at all stages of ACP and EoLC – a priority noted by Meeker et al. (2014) nearly a decade ago. In summary, the areas of feedback from the families and carers were most significantly related to: practical supports (such as where to access food); physical supports (such as the provision of resting space); and, an area stressed by participants in particular, the need for timely and respectful communication from their specialists. Finally, the families and carers involved in this study were united in their wish that all staff members see the whole person, including, as Hossain et al. (2022) suggest, the cultural and religious identity of patient, family, and carers. To give the final summation of advice to staff in the words of one of the participants, reflecting a desire for whole of person (including spiritual needs) treatment, it is this: “*don’t lose track of the soul.*”
